# Albuminuria Increases All-Cause Mortality in Japanese Patients with Type 2 Diabetes Mellitus

**DOI:** 10.3390/jcm7090234

**Published:** 2018-08-23

**Authors:** Hitomi Miyake, Ippei Kanazawa, Toshitsugu Sugimoto

**Affiliations:** Department of Internal Medicine 1, Shimane University Faculty of Medicine, Izumo 693-8501, Japan; hito@med.shimane-u.ac.jp (H.M.); sugimoto@med.shimane-u.ac.jp (T.S.)

**Keywords:** albuminuria, mortality, eGFR, type 2 diabetes mellitus

## Abstract

Previous studies have reported that diabetic kidney disease is associated with cardiovascular events and death. Little is known about the independent association of albuminuria and estimated glomerular filtration rate (eGFR), with mortality in Asian patients with type 2 diabetes mellitus (T2DM) without renal failure. We conducted a historical cohort study to clarify this issue in Japanese patients with T2DM. In this study, we recruited 385 patients with T2DM, who never had chronic renal failure (eGFR < 30 mL/min/1.73 m^2^ at baseline) and malignant diseases. With the end point of all-cause mortality, Cox regression analysis was performed. During the observational period of 7 years, 54 patients died. Cox regression analysis adjusted for confounding factors such as age, duration of diabetes, body mass index, and HbA1c, and showed that urinary albumin level was significantly associated with the mortality [hazard ratio (HR) = 1.32, 95% confidence interval (CI) = 1.03–1.70 per standard deviation (SD) increase, *p* = 0.031]. After additional adjustment for eGFR, the association remained significant (HR = 1.32, 95% CI = 1.02–1.70 per SD increase, *p* = 0.033). On the other hand, eGFR was not associated with the mortality. The present study showed that higher urinary albumin was associated with increased all-cause mortality in T2DM, independently of eGFR. These findings suggest that, regardless of eGFR, albuminuria is important for the increased risk of mortality in Japanese T2DM patients without chronic renal failure (eGFR < 30 mL/min/1.73 m^2^). However, because of several limitations, further large-scale longitudinal studies are necessary to confirm the present study.

## 1. Introduction

The number of patients with diabetes mellitus (DM) is rapidly increasing worldwide, especially in Asian countries. Chronic kidney disease (CKD) is more common in subjects with DM than those without it, and DM is already the leading cause of CKD and end-stage kidney disease (ESKD) [1,2]. It has been shown that CKD is an independent risk factor, not only for ESKD, but also cardiovascular disease (CVD) and all-cause mortality in patients with DM [3,4]. Previous studies have shown that the adjusted relative risk of death increased up to twice in patients with DM, compared to age-matched controls [5,6]. Therefore, it is very important to determine and manage diabetic kidney disease, to protect against progression of renal dysfunction and CVD, and prolong their life expectancy.

Accumulating evidence has shown that increasing albuminuria and decreased estimated glomerular filtration rate (eGFR), are significant risk factors for cardiovascular and all-cause mortality, not only in Western countries, but also Asian [7,8,9,10,11]. A prospective cohort study in multi-ethnic Asian populations involving Malay, Chinese, and Indian adults, previously showed that the risks of CVD and all-cause mortality were significantly increased with albuminuria and eGFR reduction [11]. In contrast, several studies suggested that the association between CKD and mortality might be different between Asian and Western patients with DM [12,13]. Li et al. recently reported that the incidence of all-cause mortality, cardiovascular death, and major coronary events was significantly lower in Asian patients than in Western patients [12]. In contrast, the incidence of major cerebrovascular events, microvascular events, and nephropathy was significantly higher in Asian patients than in Western patients. Moreover, several genetic factors are involved in the ethnic differences in diabetic kidney disease [14,15]. For example, polymorphism of the angiotensin-converting enzyme gene is associated with progression to ESKD in Asian patients, but this association is uncertain in Western patients [14]. Furthermore, Liu et al. showed that there are differences in the risk of CVD and ESKD among native Asian subpopulations with type 2 diabetes mellitus (T2DM) [13]. Therefore, it is important to confirm the association of CKD with vascular events and mortality, in individual countries.

Anti-diabetic and renal protective agents, as well as management strategies for diabetes and CKD, have been developed. In addition, many physicians have recently paid attention to metabolic diseases, including DM and CKD. However, in Japan, the number of diabetic patients with ESKD is still increasing, and a recent study using a survey of hospital records during 2001–2010, reported that the average lifespan of patients with DM is shorter than that of general population [16]. Therefore, it is important task to determine the risk factor for all-cause mortality, and the association of albuminuria and eGFR, with the risk of mortality in Japanese patients with DM. Wada et al. previously reported a historical cohort study of Japanese patients with T2DM, showing that increased urinary albumin-to-creatinine ratio levels were closely associated with the increase in risks for renal and cardiovascular events, as well as all-cause mortality, whereas eGFR was not associated in cases of patients with eGFR over 30 mL/min/1.73 m^2^ [17]. These findings suggest that the association between renal function and mortality in Japanese patients, seems to be different from previous studies performed using other ethnic populations [7,8,9,10,11]. To confirm the association between albuminuria and eGFR, with all-cause mortality in Japanese patients with T2DM, we conducted a historical cohort study.

## 2. Materials and Methods

### 2.1. Subjects

This was a historical cohort study investigating the association between diabetic kidney disease and the endpoint of all-cause mortality, in patients with T2DM. The patients who were admitted to Shimane University Hospital, for education and treatment of T2DM from 1997–2009, were screened. We consecutively assessed the presence of diabetic kidney disease in patients with T2DM, who were admitted to our hospital, for the treatment of T2DM, except for having malignant diseases, infection, necessity of operation, and other special purposes. Out of them, there were 698 patients with T2DM, whose daily urinary albumin excretion and eGFR were measured on admission. We excluded 5 patients with chronic renal failure (eGFR < 30 mL/min/1.73 m^2^), because of the small number. We investigated patient survival or mortality, using medical records and telephone surveys from 2013 to 2014, with a median follow-up period of 7 years. Unfortunately, we were unable to contact 308 patients. Finally, 385 patients with T2DM were included in this study. This study was approved by the institutional review board of Shimane University Faculty of Medicine; the requirement for informed patient consent was waived because no intervention and further examinations were performed.

### 2.2. Biochemical Measurements

After overnight fasting, blood samples were collected. Urinary albumin excretion levels were estimated by 24 h urine collection. Biochemical markers were measured by standard methods, as previously described in References [18,19]. HbA1c was determined using high-performance liquid chromatography. HbA1c values were estimated as NGSP (National Glycohemoglobin Standardization Program) equivalent values, calculated by the formula: HbA1c (%) = HbA1c (JDS) (Japan Diabetes Society) (%) + 0.4%. eGFR was calculated using the equation proposed by the Modification of Diet in Renal Disease Study, with modified coefficients for Japanese, according to Reference [20].

### 2.3. Statistical Analysis

Data were expressed as means ± standard deviation (SD). Given that the duration of diabetes and urinary albumin showed markedly skewed distributions, these were expressed as a median (interquartile rage), and logarithmic transformation (log) was carried out before performing correlation analysis. Statistical evaluations for differences between two groups were carried out using Student’s *t* test for parametric variables, or Mann-Whitney U test for non-parametric variables. Cox proportional hazard regression models were used to estimate the risk of mortality, in a model adjusted for confounding factors. Statistical analyses were performed using a statistical computer program StatView (Abacus Concepts, Berkeley, CA, USA). A *p* value < 0.05 was considered to be significant.

## 3. Results

### 3.1. Baseline Characteristics of Subjects and Comparison of Various Parameters Between Dead Patients and Survivors

Baseline characteristics, such as biochemical and demographic parameters, are shown in Table 1. We compared these parameters between dead patients and survivors. During the observational period of 7 years, 54 patients died. Dead patients were significantly older (*p* < 0.001) and had longer duration of diabetes (*p* = 0.038). Body mass index (BMI) and eGFR were significantly lower (*p* = 0.005 and *p* = 0.027, respectively) in dead patients, than those in survivors. Urinary albumin levels were not different between them (*p* = 0.265).

### 3.2. Correlation of Urinary Albumin and eGFR with Various Variables

We performed simple correlation analysis to examine the relationship between urinary albumin and eGFR, versus various parameters (Table 2). Urinary albumin, was significantly and positively correlated with BMI (*r* = 0.12, *p* = 0.023), fasting plasma glucose (FPG) (*r* = 0.18, *p* < 0.001), and HbA1c (*r* = 0.19, *p* < 0.001). eGFR, was significantly and positively correlated with FPG (*r* = 0.19, *p* < 0.001) and HbA1c (*r* = 0.12, *p* = 0.019), and negatively with age (*r* = −0.43, *p* < 0.001) and duration of diabetes (*r* = −0.21, *p* < 0.001).

### 3.3. Association of Urinary Albumin and eGFR with All-Cause Mortality

Because age, duration of diabetes, BMI, and HbA1c were significantly related to urinary albumin, eGFR, and mortality, we performed Cox regression analyses adjusted for these confounding parameters, to evaluate the associations of albuminuria and eGFR reduction with all-cause mortality (Table 3). In the Cox regression analysis adjusted for age (model 1), urinary albumin level was significantly associated with the risk of all-cause mortality [hazard ratio (HR) = 1.31, 95% confidence interval (CI) = 1.03–1.37, *p* = 0.031]. After additional adjustment for HbA1c, BMI, and duration of diabetes (model 4), the association between urinary albumin and all-cause mortality remained significant (HR = 1.32, 95% CI = 1.03–1.70, *p* = 0.031). Moreover, even after adjusting for eGFR (model 5), the association still remained significant (HR = 1.32, 95% CI = 1.02–1.70, *p* = 0.033). In contrast, eGFR was not associated with the risk of all-cause mortality (model 1 to 4 and 6).

## 4. Discussion

The present study showed that albuminuria was significantly associated with the increased risk of all-cause mortality in Japanese patients with T2DM, whereas eGFR was not. These findings were consistent with the previous report by Wada et al. [17]. Moreover, the association between albuminuria and all-cause mortality was independent of eGFR. Therefore, albuminuria is an important predictor of all-cause mortality in Japanese patients with T2DM, who had eGFR over 30 mL/min/1.73 m^2^.

Numerous studies have shown that decreased eGFR, even in CKD stage 2 and 3, is associated with mortality in patients with DM [8,9,10,11]. It is unclear why eGFR was not associated with all-cause mortality in Japanese cohort studies. We analyzed only subjects who visited Shimane University Hospital, a tertiary center for treatment of diabetes mellitus. Furthermore, the previous study reported by Wada et al. [17] included participants who were treated by trained physicians, at the diabetes and renal division. Therefore, the participants enrolled in this and previous studies might have had relatively severe states of the disorder and might not have been representative of Japanese patients with the disorder. Furthermore, the information regarding the treatments for diabetes and CKD was not available during the observation; thus, treatments such as diet therapy and medications might affect the association between eGFR and mortality. 

On the other hand, serum creatinine levels are known to be affected by muscle mass, diet, age, and race [21]. Thus, estimation using other parameters, such as cystatin C, might be useful for the prediction of mortality risk in Japanese. Indeed, Ide et al. have recently shown that eGFR calculated by serum cystatin C is a better predictor for all-cause mortality than eGFR calculated by serum creatinine, in Japanese patients with T2DM [22]. In their study, compared to creatinine-based eGFR ≥ 90 mL/min/1.73 m^2^, creatinine-based eGFR < 30 mL/min/1.73 m^2^ was significantly associated with increased risk of all-cause mortality, although creatinine-based eGFR 60–89 mL/min/1.73 m^2^ and 30–59 mL/min/1.73 m^2^ were not associated. In contrast, cystatin C-based eGFR 30–59 mL/min/1.73 m^2^ and <30 mL/min/1.73 m^2^, were significantly associated with all-cause mortality. Taken together, creatinine-based eGFR may not be a useful marker for prediction of all-cause mortality risk, in Japanese T2DM patients, without <30 mL/min/1.73 m^2^.

Some limitations of the present study should be considered. First, the sample size was not large enough to make definite conclusions. Although the lack of an association between eGFR and all-cause mortality might be due to a limited number of the subjects, the estimated hazard ratio (around 1.00) implied that no significant association would be expected even if the sample size were increased. Second, we could not contact many of the patients to verify if they were alive or dead. Therefore, there is a possibility that we could not contact them because of death. Third, we could not include many confounding factors of albuminuria, eGFR, and mortality. Fourth, histological evidence for diabetic kidney disease was lacking in this study. Finally, we could not analyze the cause of death, such as cardiovascular, infection, and malignant diseases.

## 5. Conclusions

In conclusion, the present study showed that higher urinary albumin levels were associated with the increased all-cause mortality in Japanese patients with T2DM, independently of eGFR. These findings suggest that, regardless of eGFR, albuminuria is important for the increased risk of mortality in Japanese T2DM patients without chronic renal failure. Moreover, serum creatinine-based eGFR was not good marker for all-cause mortality, in the early stage of diabetic kidney disease. Thus, other renal parameters, such as cystatin C, may eventually prove to be superior to serum creatinine in Japanese. However, because there were several limitations in this study, further large-scale longitudinal studies are necessary to confirm the present findings.

## Figures and Tables

**Table 1:** Baseline characteristics of subjects and comparison of demographic and biochemical parameters between dead patients and survivors.

	Total	Alive	Dead	*p* Value
Number of subjects	385	331	54	
Age (years)	65.7 ± 9.9	64.2 ± 9.5	73.5 ± 7.9	<0.001
Duration of diabetes (years)	10.0 (1.0–18.0)	9.0 (1.0–18.0)	10.0 (5.0–20.0)	0.038
BMI (kg/m^2^)	23.9 ± 4.2	24.1 ±4.2	22.4 ± 3.6	0.005
FPG (mg/dL)	164 ± 58	164 ± 58	175 ± 62	0.222
HbA1c (%)	8.7 ± 2.1	8.7 ± 2.1	9.3 ± 2.4	0.083
C-peptide (ng/mL)	1.7 ± 0.9	1.7 ± 0.9	1.6 ± 1.1	0.225
eGFR (mL/min/1.73 m^2^)	78.5 ± 20.0	80.7 ± 19.6	74.2 ± 20.8	0.027
Urinary albumin (mg/day)	13.7 (7.3–49.4)	13.0 (7.3–48.0)	17.2 (8.4–50.6)	0.265

**Table 2:** Correlation of urinary albumin and eGFR with background characteristics.

	Log(uAlb)		eGFR	
	*r*	*p*	*r*	*p*
Age	−0.09	0.065	−0.43	<0.001
Duration of diabetes	0.09	0.100	−0.21	<0.001
BMI	0.12	0.023	0.05	0.349
FPG	0.18	<0.001	0.19	<0.001
HbA1c	0.19	<0.001	0.12	0.019
C-peptide	0.08	0.124	−0.08	0.108
eGFR	−0.01	0.907		

*r*, correlation coefficient; *p*, *p* value.

**Table 3:** Hazard ratios stratified by albuminuria or eGFR.

	HR	95% CI	*p* Value
Urinary albumin			
Model 1	1.31	1.03–1.67	0.031
Model 2	1.28	1.00–1.64	0.049
Model 3	1.29	1.01–1.66	0.045
Model 4	1.32	1.03–1.70	0.031
Model 5	1.32	1.02–1.70	0.033
eGFR			
Model 1	1.07	0.78–1.46	0.673
Model 2	1.05	0.76–1.43	0.778
Model 3	1.00	0.73–1.37	0.981
Model 4	0.95	0.69–1.31	0.952
Model 5	0.97	0.71–1.33	0.853

Cox proportional hazard regression models were performed with all-cause mortality as a dependent variable. Model 1; adjusted for age. Model 2; adjusted for model 1 plus HbA1c. Model 3; adjusted for model 2 plus BMI. Model 4; adjusted for model 3 plus duration of diabetes. Model 5; adjusted for model 4 plus eGFR. Model 6; adjusted for model 4 plus urinary albumin. Unit of change; standard deviation per increase. HR, hazard ratio; CI, confidential interval.
